# Associations between life’s essential 8 and femoral neck bone mineral density among adults: A national population-based study

**DOI:** 10.1097/MD.0000000000039540

**Published:** 2024-09-06

**Authors:** Linjian Liu, An Zhang, Xiangjun Xiao

**Affiliations:** a Hengyang Medical School, University of South China, Hengyang, China; b Department of Pharmacy, The Xiangxi Autonomous Prefecture Traditional Chinese Medicine Hospital, Jishou, China.

**Keywords:** bone mineral density, cardiovascular health, life’s essential 8, National Health and Nutrition Examination Survey, osteoporosis

## Abstract

Osteoporosis represents a significant public health issue, impacting both health outcomes and economic costs. This research investigates how cardiovascular health, as indicated by the LE8 score, correlates with bone mineral density (BMD). Data from the National Health and Nutrition Examination Survey (NHANES) spanning 2011 to 2018 were analyzed in this cross-sectional analysis, including 9018 subjects following the exclusion of individuals lacking BMD or LE8 data. The LE8 score, comprising factors such as diet, physical activity, smoking status, sleep quality, body mass index, lipid profiles, blood glucose, and blood pressure, was used to evaluate cardiovascular health. BMD was determined through dual-energy X-ray absorptiometry (DXA). Relationships between the LE8 scores and BMD at the femoral neck were assessed using linear regression and smooth curve fitting techniques. Enhanced LE8 scores were linked to improved BMD at the femoral neck. Notably, a 10-point increment in the LE8 score was associated with a rise in BMD by 0.04 g/cm² [β = 0.04, 95% CI: 0.03–0.05]. The data indicate a strong positive association between cardiovascular health, as measured by LE8, and BMD. These results support the development of holistic health strategies that promote cardiovascular health to potentially improve bone density.

## 1. Introduction

Osteoporosis, identified as a widespread skeletal disorder, is characterized by reduced bone mass and deterioration of bone structure, posing a significant public health concern predominantly among older adults and postmenopausal women.^[1]^ Globally, osteoporosis leads to approximately 8.9 million fractures annually, translating to a fracture due to osteoporosis nearly every 3 seconds.^[2,3]^ This condition significantly heightens the likelihood of fractures, especially in the hip, spine, and wrist areas. In the United States, it is estimated that osteoporosis affects around 10 million people, with an additional 44 million exhibiting low bone mass.^[4]^ The economic impact of osteoporosis is substantial, reflecting in the high costs associated with fracture treatments and their chronic complications, including prolonged disability and elevated mortality rates.^[5,6]^

Cardiovascular health (CVH) plays an essential role in overall well-being and life expectancy, involving key elements like blood pressure, cholesterol levels, glucose levels, physical activity, dietary habits, body weight, and smoking status.^[7]^ The American Heart Association (AHA) underscores the significance of these components through the life’s simple 7 score, which evaluates cardiovascular risks based on 7 adjustable behaviors and health factors.^[8]^ Studies have shown that adherence to ideal cardiovascular metrics significantly decreases the occurrence of cardiovascular diseases (CVDs), the primary cause of death worldwide. Optimal CVH is also associated with a lower risk of other chronic diseases, highlighting its extensive impact on public health.^[9,10]^

Recent studies have unveiled a significant correlation between CVH and bone mineral density (BMD).^[11,12]^ Cardiovascular risk factors such as diabetes, hypertension, and elevated cholesterol levels are suspected to negatively affect bone health. Possible underlying mechanisms may include inflammation and vascular calcification, which impact both cardiovascular and bone tissues.^[13–15]^ Moreover, lifestyle choices critical to CVH, like regular exercise and healthy eating, directly contribute to bone density.^[16,17]^ This research seeks to further investigate these connections by examining the relationship between the LE8 CVH score and BMD in adults. By understanding how CVH influences bone density, we aim to refine prevention strategies for both osteoporosis and cardiovascular conditions, thereby improving collective health outcomes.^[18]^

## 2. Methods

### 2.1. Study population

**Figure 1.**
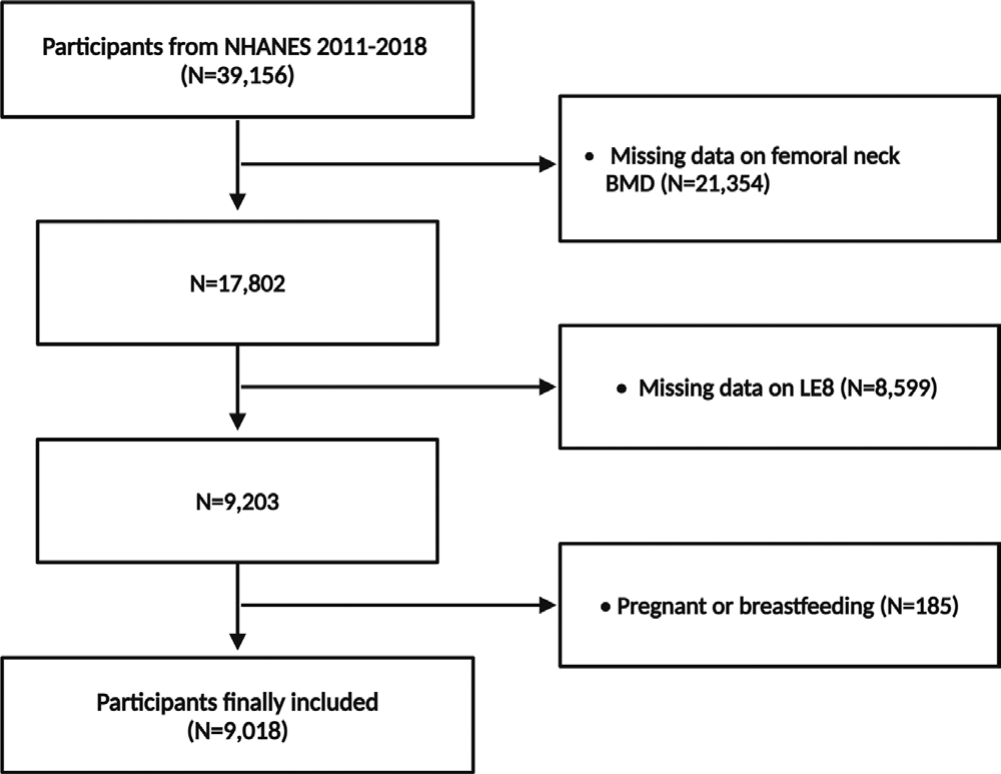
Flow chart of participant’s selection. NHANES, National Health and Nutrition Examination Survey.

This study utilizes data from the National Health and Nutrition Examination Survey (NHANES) covering the period from 2011 to 2018, as executed by the National Center for Health Statistics. NHANES assesses the health and nutritional status of Americans through a sophisticated, multi-stage, probability sampling design ensuring a nationally representative sample.^[19,20]^ Ethics approval was granted by the NCHS Research Ethics Review Board, and all participants provided informed consent.^[21]^ From the initial dataset comprising 39,156 participants, exclusions were made for those lacking femoral neck BMD data (N = 21,354), incomplete LE8 scores (N = 8599), and women who were pregnant or breastfeeding (N = 185), resulting in a study cohort of 9018 adults aged 20 years and older (Fig. 1).

### 2.2. Study variables

The primary variable of interest, femoral neck BMD, was measured using sector beam densitometry through the Hologic APEX v4.0 software, alongside Dual-Energy X-ray Absorptiometry (DXA) techniques from Shepherd laboratory.^[22]^ Affirmative responses to either question classified participants as having infertility. Cardiovascular health is evaluated through the LE8 score, which includes diet (aligned with the Dietary Approaches to Stop Hypertension (DASH) guidelines), physical activity, tobacco use, sleep, body mass index (BMI), lipid levels excluding high-density lipoprotein (HDL), blood glucose, and blood pressure. Standard surveys collect lifestyle data, while physical and biochemical measurements are taken following standardized methods. BMI is calculated as weight in kilograms divided by height in meters squared. Blood pressure is averaged from initial readings, non-HDL cholesterol is obtained by subtracting HDL from total cholesterol, and glycated hemoglobin is determined by liquid chromatography (detailed in Table S1, Supplemental Digital Content, http://links.lww.com/MD/N526). The LE8 score ranges from 0 to 100, with an aggregate score derived from these components, categorizing LE8 into high, moderate, or low according to AHA benchmarks.^[7,23]^ Covariates included age, race, educational level, and the ratio of family income-to-poverty (PIR), chosen for their epidemiological significance and potential confounding effects.^[24]^

### 2.3. Statistical analysis

The statistical analyses were conducted using R software, version 4.3.0, and all significance tests were 2-tailed with a threshold of *P* < .05. To handle missing data, we employed multiple imputation via the mice package, generating ten separate imputation datasets to ensure robust estimates. Cardiovascular health was categorized into 3 distinct LE8 score groups: low (below 50), intermediate (50–79), and high (80 or above).

We used linear regression models to evaluate the relationship between cardiovascular health and BMD. Three models were constructed for this purpose: model 1: unadjusted; model 2: adjusted for age and ethnicity; model 3: additionally adjusted for educational attainment and PIR.

To investigate potential nonlinear associations between LE8 scores and BMD, we utilized generalized additive models. These models provide flexibility by allowing the data to determine the shape of the relationship, which is optimized using the generalized cross-validation score.

Furthermore, we conducted interaction analyses to explore whether the LE8–BMD relationship differed among various subgroups. This was done by incorporating interaction terms into our models, representing the interaction between LE8 scores and subgroup identifiers. These analyses allowed us to assess the heterogeneity of the LE8–BMD association across different demographic and socioeconomic strata.

## 3. Results

### 3.1. Baseline characteristics

The study included 9018 adult participants with an average age of 56.2 years (SD = 18.2). The cohort comprised 51.1% male and 48.9% female participants. The mean LE8 score was 67.6 (SD = 11.3), and the average femoral neck BMD was 1.04 g/cm² (SD = 0.2). Notably, 16.3% of participants scored low on LE8 (LE8 < 50), 63.0% had moderate scores (50 ≤ LE8 < 80), and 20.7% scored high (LE8 ≥ 80). Higher LE8 scores correlated with increased femoral neck BMD. Furthermore, participants with higher LE8 scores tended to be younger, female, and of higher socioeconomic status, as indicated by educational attainment and income-to-poverty ratios (*P* < .01) (Table 1).

**Table 1:** Baseline characteristics of participants with different CVH levels estimated from LE8 score.

Characteristics	Low (LE8 < 50)	Moderate (50 ≤ LE8 < 80)	High (LE8 ≥ 80)	*P* value
No. of participants in sample	1488	5932	1598	
Age, y (SD)	55.17 ± 11.91	43.55 ± 11.53	39.05 ± 11.02	<.001
PIR	2.05 ± 1.48	2.56 ± 1.61	3.03 ± 1.71	
Lumbar BMD, g/cm^2^ (SD)	1.03 ± 0.16	1.03 ± 0.15	1.06 ± 0.14	<.001
Gender, %				<.001
Male	54.92	53.18	42.77	
Female	45.08	46.82	57.23	
Race/ethnicity, %				<.001
Non-Hispanic White	39.76	35.08	38.88	
Non-Hispanic Black	28.08	22.81	12.65	
Mexican American	14.37	15.19	11.04	
Other Hispanic	9.51	16.69	27.94	
Others	8.27	10.23	9.49	
Education level, %				<.001
Less than high school	27.17	17.54	7.41	
High school	28.74	22.91	12.96	
More than high school	44.09	59.55	79.63	
AHA LE8 score (SD)				
Total CVH score	41.54 ± 6.48	65.00 ± 8.22	87.06 ± 5.36	<.001
Mean DASH diet score	22.29 ± 25.04	38.75 ± 30.76	64.43 ± 29.14	<.001
Mean physical activity score	12.41 ± 30.45	48.41 ± 47.07	89.38 ± 26.17	<.001
Mean tobacco/nicotine exposure score	37.88 ± 38.58	66.92 ± 38.94	90.62 ± 21.64	<.001
Mean sleep health score	67.02 ± 30.62	82.09 ± 24.07	91.51 ± 16.42	<.001
Mean body mass index score	32.81 ± 29.54	58.24 ± 33.24	85.30 ± 21.92	<.001
Mean blood lipid score	46.09 ± 30.03	66.75 ± 30.00	87.42 ± 21.66	<.001
Mean blood glucose score	65.03 ± 31.80	86.27 ± 23.23	97.62 ± 9.80	<.001
Mean blood pressure score	48.80 ± 29.52	72.57 ± 28.17	90.23 ± 18.35	<.001

Mean (SD) for continuous variables: the *P* value was calculated by the weighted linear regression model.

Percentages for categorical variables: the *P* value was calculated by the weighted chi-square test.

Cardiovascular health (CVH) is categorized into 3 grades, low: LE8 score < 50, medium: 50 ≤ LE8 score < 80, high: LE8 score ≥ 80.

Abbreviations: AHA = American Heart Association, BMD = bone mineral density, CVH = cardiovascular health, DASH = dietary approaches to stop hypertension, LE8 = life’s essential 8, PIR = the ratio of family income to poverty.

### 3.2. Association between LE8 and femoral neck BMD

Table 2 outlines the relationship between the LE8 scores and femoral neck BMD. Across all models, a significant positive correlation was observed between LE8 scores and BMD (*P* < .05). In the fully adjusted model, each 10-point increase in the LE8 score corresponded to a 0.04 g/cm² increase in BMD [β = 0.04, 95% CI: 0.03–0.05]. Participants in the high LE8 category exhibited a 0.02 g/cm² higher BMD than those in the low category [β = 0.03, 95% CI: 0.02–0.04]. There was no notable difference in BMD between participants with moderate and low LE8 scores [β = −0.00, 95% CI: −0.01 to 0.01].

**Table 2:** The association between the life’s essential 8 and femoral neck BMD.

Lung function indices	Model 1 [β (95% CI)]	Model 2 [β (95% CI)]	Model 3 [β (95% CI)]
Total CVH score (per 10 scores)	0.05 (0.04, 0.06)	0.04 (0.03, 0.05)	0.04 (0.03, 0.05)
CVH categories			
Low (LE8 < 50)	0	0	0
Moderate (50 ≤ LE8 < 80)	−0.00 (−0.01, 0.01)	0.00 (−0.01, 0.01)	0.00 (−0.01, 0.01)
High (LE8 ≥ 80)	0.03 (0.01, 0.04)	0.03 (0.02, 0.04)	0.03 (0.02, 0.04)
*P* for trend	.160	.123	.101

Model 1 was unadjusted for covariates; model 2 enhanced model 1 by including age, sex, and ethnicity; and model 3 further augmented model 2 by integrating education level and family income-to-poverty ratio.

Abbreviation: CVH = cardiovascular health.

Table 3 shows consistent positive associations between LE8 scores and femoral neck BMD across various demographic subgroups, including sex, age, education, and income-to-poverty ratio, with no significant interactions (all *P* for interaction > .05).

**Table 3:** Subgroup analysis of the association between LE8 (per 10 scores) and femoral neck BMD.

Subgroup	Femoral neck BMD [β (95%CI)]	*P* for interaction
Sex		.559
Male	0.04 (0.02, 0.06)	
Female	0.03 (0.01, 0.05)	
Age		.135
<60 yr	0.03 (0.01, 0.05)	
≥60 yr	0.03 (0.02, 0.04)	
Education level		.904
Less than high school	0.03 (0.01, 0.06)	
High school	0.02 (−0.01, 0.05)	
More than high school	0.03 (0.01, 0.05)	
PIR		.116
<1.3	0.02 (−0.03, 0.06)	
1.3 to 3.5	0.03 (0.01, 0.05)	
>3.5	0.02 (0.00, 0.04)	

Age, sex, race, education level, and PIR. Abbreviation: BMD = bone mineral density, CVH = cardiovascular health, PIR = the ratio of income to poverty.

## 4. Discussion

Our research, which encompassed 9018 U.S. adults, examined the correlation between a comprehensive cardiovascular health index (LE8 score) and BMD. We found that higher LE8 levels are associated with increased femoral neck BMD, where each 10-point increment in the LE8 score corresponded to a 0.04 g/cm² increase in BMD. These findings underscore the importance of managing health behaviors and factors to maintain cardiovascular health, which may be crucial for healthy bone metabolism and reducing osteoporosis prevalence.

### 4.1. Comparison with previous studies

To our knowledge, this is the inaugural study to assess the relationship between LE8 and BMD using a quantifiable cardiovascular health indicator. Earlier studies typically focused on isolated cardiovascular or bone health factors, not a holistic index.^[14,25,26]^ For instance, earlier research identified negative correlations between apolipoprotein B levels and femoral neck BMD, indicating that higher levels of this lipid-related protein could be detrimental to bone density.^[15]^

Emerging evidence suggests that lipid profiles, when considered as part of broader cardiovascular health metrics, may influence bone density at various skeletal sites. Specifically, higher lipid levels have been associated with lower bone density, suggesting a potential adverse effect on bone health.^[13,27]^ Supporting this, a study utilizing NHANES data observed a negative relationship between total cholesterol levels and total BMD, reinforcing the notion that cholesterol and other cardiovascular health metrics are integral to bone health.^[28]^

More direct associations between cardiovascular diseases and BMD have been documented; for instance, 1 study noted that femoral bone density has a nonlinear relationship with cardiovascular disease risk, showing that as BMD decreases, cardiovascular risk increases, particularly once a certain threshold is surpassed.^[29]^ Another study reported lower BMD associated with an increased risk of cardiovascular events, specifically in Asian women, suggesting that lower bone density could predict adverse cardiovascular outcomes.^[30]^ Our study advances these findings by integrating a range of cardiovascular health markers into a single LE8 score, providing a more comprehensive view of how overall cardiovascular health impacts BMD. This approach not only corroborates the links identified in previous studies but also enhances our understanding by showing how a composite health index might be predictive of bone health. This suggests the potential for using cardiovascular health improvement strategies as part of interventions aimed at maintaining or improving BMD.

### 4.2. Potential biological mechanisms

The positive association between LE8 and BMD can be explained through the complex interactions of various lifestyle and physiological factors included in the LE8 score. These factors – such as diet, physical activity, tobacco use, sleep, BMI, lipid levels, blood glucose, and blood pressure – contribute to both cardiovascular and bone health through distinct yet interrelated mechanisms. For example, diets aligned with DASH guidelines, which are rich in calcium and low in sodium, support bone health by optimizing calcium metabolism, essential for bone maintenance and growth.^[16]^ Physical activity promotes bone formation and density through mechanical stress-induced bone remodeling.^[31]^ Conversely, smoking impairs calcium absorption and disrupts hormonal balance, negatively impacting bone integrity.^[32]^ Sleep quality affects hormonal regulation crucial for bone health, with disruptions linked to lower BMD and increased fracture risks.^[17]^ BMI impacts bone density differently at various levels; a higher BMI generally correlates with greater bone mass due to increased mechanical load, although extremes in BMI can adversely affect bone structure.^[33,34]^ Elevated lipid levels, particularly cholesterol, have been associated with increased bone resorption through inflammatory pathways.^[35]^ High blood glucose and insulin resistance impair bone quality and decrease formation rates, underscoring the importance of managing diabetes for bone health.^[29]^ Lastly, hypertension affects bone health by altering calcium and phosphate metabolism, making effective blood pressure management a critical component for maintaining bone density.^[36]^ Understanding these multifactorial interactions helps in developing comprehensive strategies to manage and potentially improve both cardiovascular and skeletal health

### 4.3. Strengths and limitations

Our study boasts several strengths and faces certain limitations that shape the interpretation and applicability of our findings. Among the strengths, the use of the NHANES dataset, which is a large, nationally representative sample, provides robust statistical power and enhances the generalizability of our results across the U.S. adult population. Additionally, the comprehensive nature of the LE8 index, which integrates multiple cardiovascular and lifestyle factors, allows for a nuanced analysis of their collective impact on BMD.^[37]^

However, our study also has limitations. The cross-sectional design limits our ability to infer causal relationships between LE8 and BMD.^[38]^ Longitudinal studies would be required to confirm the directionality and persistence of these associations over time. Moreover, while NHANES includes a diverse range of participants, the findings may not be fully generalizable to populations outside the United States or to specific subgroups not well represented in the survey.^[39]^ Another potential limitation is the reliance on self-reported data for some of the cardiovascular and lifestyle measures, which can introduce recall bias and affect the accuracy of the associations observed.^[40]^ Furthermore, although we adjusted for a wide array of confounders, residual confounding by unmeasured or inaccurately measured variables could still influence the outcomes. Future studies might benefit from incorporating more direct measures of cardiovascular health and bone density, as well as exploring the potential mediating effects of novel biomarkers and genetic factors on the relationship between LE8 and BMD.

## 5. Conclusion

This study demonstrated a significant positive correlation between LE8 levels and femoral neck BMD using cross-sectional data from U.S. adults in the NHANES database. Specifically, higher LE8 scores were associated with increased femoral neck BMD, with an average increase of 0.03 g/cm² for each 10-point increment in the LE8 score.

These findings highlight the clinical importance of maintaining elevated cardiovascular health to support healthy bone metabolism and reduce the risk of osteoporosis. Clinically, this suggests that improving cardiovascular health through lifestyle changes or medical interventions could also benefit bone health. Healthcare providers should consider comprehensive cardiovascular health assessments in routine evaluations for patients at risk of osteoporosis, integrating cardiovascular and bone health strategies to enhance overall patient care.

## Acknowledgments

Our gratitude extends to every participant involved in this study.

## Author contributions

**Conceptualization:** Linjian Liu, An Zhang, Xiangjun Xiao.

**Data curation:** Linjian Liu, An Zhang, Xiangjun Xiao.

**Formal analysis:** Linjian Liu, An Zhang, Xiangjun Xiao.

**Funding acquisition:** Xiangjun Xiao.

**Investigation:** Linjian Liu, An Zhang, Xiangjun Xiao.

**Methodology:** Linjian Liu, An Zhang, Xiangjun Xiao.

**Project administration:** Linjian Liu, Xiangjun Xiao.

**Resources:** Linjian Liu, Xiangjun Xiao.

**Software:** Linjian Liu, An Zhang, Xiangjun Xiao.

**Supervision:** Linjian Liu, An Zhang, Xiangjun Xiao.

**Validation:** Linjian Liu, An Zhang, Xiangjun Xiao.

**Visualization:** Linjian Liu, Xiangjun Xiao.

**Writing – original draft:** Linjian Liu, An Zhang, Xiangjun Xiao.

**Writing – review & editing:** Linjian Liu, Xiangjun Xiao.

## Supplementary Material


