# Supplementary material for: Associations between life’s essential 8 and femoral neck bone mineral density among adults: A national population-based study
Source: Medicine (Baltimore). 2024 Sep 6;103(36):e39540. doi: 10.1097/MD.0000000000039540 (PMC11384864; doi:10.1097/MD.0000000000039540)
Supplement: Supplementary file 1 [file medi-103-e39540-s001.pdf]

Table S1. Definition and scoring approach for quantifying cardiovascular health, as per the American Heart Association's Life's Essential 8 score, and as applied in the National Health and Nutrition Examination Surveys.

Domain	CVH Metric	Method of Measurement	Quantification of CVH Metric - Adults (≥20 Years)	Quantification of CVH Metric – Children (Up to 19 Years*)
Health Behaviors	**Diet**	**Measurement**: Self-reported daily intake of a DASH-style eating pattern<br><br>**Example tools for measurement:** DASH diet score (populations)	Quantiles of DASH-style diet adherence<br><br>**Scoring (Population):**<br>Points Quantile<br>100 ≥95th %ile (top/ideal diet)<br>80 75th – 94th %ile<br>50 50th – 74th %ile<br>25 25th – 49th %ile<br>0 1st – 24th %ile (bottom/least ideal quartile)	Quantiles of DASH-style diet adherence; ages 2-19<br><br>**Scoring (Population):**<br>Points Quantile<br>100 ≥95th %ile (top/ideal diet)<br>80 75th – 94th %ile<br>50 50th – 74th %ile<br>25 25th – 49th %ile<br>0 1st – 24th %ile (bottom/least ideal quartile)
	**Physical activity**	**Measurement:** Self-reported minutes of moderate or vigorous physical activity per week<br><br>**Example tools for measurement:**<br>NHANES PAQ-K questionnaire	**Metric:** Minutes of moderate (or greater) intensity activity per week<br><br>**Scoring:**<br>Points Minutes<br>100 ≥150<br>90 120 – 149<br>80 90 – 119<br>60 60 – 89<br>40 30 – 59<br>20 1 – 29<br>0 0	**Metric:** Minutes of moderate (or greater) intensity activity per week; ages 2-19 years<br><br>**Scoring:**<br>Points Minutes<br>100 ≥420<br>90 360 – 419<br>80 300 – 359<br>60 240 – 299<br>40 120 – 239<br>20 1 – 119<br>0 0
	**Nicotine exposure**	**Measurement:** Self-reported use of cigarettes or inhaled nicotine-delivery system	**Metric:** Combustible tobacco use and/or inhaled NDS use; or secondhand smoke exposure	**Metric:** Combustible tobacco use and/or inhaled NDS use, or secondhand smoke exposure; ages 12-19

		**Example tools for measurement:**<br>NHANES SMQ	**Scoring:**<br><u>Points Status</u><br>100 Never smoker<br>75 Former smoker, quit ≥5 yrs<br>50 Former smoker, quit 1 - <5 yrs<br>25 Former smoker, quit <1 year, or currently using inhaled NDS<br>0 Current smoker<br><br>Subtract 20 points (unless score is 0) for living with active indoor smoker in home	**Scoring:**<br><u>Points Status</u><br>100 Never tried<br>50 Tried any nicotine product, but >30 days ago<br>25 Currently using inhaled NDS<br>0 Current combustible use (any within 30 days)<br><br>Subtract 20 points (unless score is 0) for living with active indoor smoker in home
	**Sleep health**	**Measurement:** Self-reported average hours of sleep per night<br><br>**Example tools for measurement:**<br>"On average, how many hours of sleep do you get per night?" Consider objective sleep/actigraphy data from wearable technology, if available	**Metric:** Average hours of sleep per night<br><br>**Scoring:**<br><u>Points Level</u><br>100 7 – <9<br>90 9 - <10<br>70 6 - <7<br>40 5 - <6 or ≥10<br>20 4 - <5<br>0 <4	**Metric:** Average hours of sleep per night; ages 16-19<br><br>**Scoring:**<br><u>Points Level</u><br>100 Age-appropriate optimal range<br>90 <1 hr above optimal range<br>70 <1 hr below optimal range<br>40 1 - <2 hrs below or ≥1 hr above optimal<br>20 2 - <3 hrs below optimal range<br>0 ≥3 hrs below optimal range
**Health Factors**	**Body mass index**	**Measurement:** Body weight (kg) divided by height squared (m^2)<br><br>**Example tools for measurement:**<br>Objective measurement of height and weight	**Metric:** Body mass index (kg/m^2)<br><br>**Scoring:**<br><u>Points Level</u><br>100 <25<br>70 25.0 – 29.9<br>30 30.0 – 34.9<br>15 35.0 – 39.9<br>0 ≥40.0	**Metric:** BMI percentiles (%iles) for age and sex; ages 2-19<br><br>**Scoring:**<br><u>Points Level</u><br>100 5th -- <85th %ile<br>70 85th -- <95th %ile<br>30 95th %ile -- <120% of the 95th %ile<br>15 120% of the 95th %ile -- <140% of the 95th %ile<br>0 ≥140% of the 95th %ile

	**Blood lipids**	**Measurement:** Plasma total and HDL-cholesterol with calculation of non-HDL-cholesterol<br><br>**Example tools for measurement:**<br>Fasting or non-fasting blood sample	**Metric:** Non-HDL-cholesterol (mg/dL)<br><br>**Scoring:**<br><u>Points Level</u><br>100 <130<br>60 130 – 159<br>40 160 – 189<br>20 190 – 219<br>0 ≥220<br><br>If drug-treated level, subtract 20 points	**Metric:** Non-HDL cholesterol (mg/dL); ages 6-19<br><br>**Scoring:**<br><u>Points Level</u><br>100 <100<br>60 100 - 119<br>40 120 - 144<br>20 145 - 189<br>0 ≥190<br><br>If drug-treated level, subtract 20 points
	**Blood glucose**	**Measurement:** Fasting blood glucose or casual hemoglobin A1c<br><br>**Example tools for measurement:**<br>Fasting (FBG, HbA1c) or non-fasting (HbA1c) blood sample	**Metric:** Fasting blood glucose (mg/dL) or Hemoglobin A1c (%)<br><br>**Scoring:**<br><u>Points Level</u><br>100 No history of diabetes and FBG <100 (or HbA1c < 5.7)<br>60 No diabetes and FBG 100 – 125 (or HbA1c 5.7-6.4) (Pre-diabetes)<br>40 Diabetes with HbA1c <7.0<br>30 Diabetes with HbA1c 7.0 – 7.9<br>20 Diabetes with HbA1c 8.0 – 8.9<br>10 Diabetes with Hb A1c 9.0 – 9.9<br>0 Diabetes with HbA1c ≥10.0	**Metric:** Fasting blood glucose (mg/dL) or Hemoglobin A1c (%); ages 12-19<br><br>**Scoring:**<br><u>Points Level</u><br>100 No history of diabetes and FBG <100 (or HbA1c < 5.7)<br>60 No diabetes and FBG 100 – 125 (or HbA1c 5.7-6.4) (Pre-diabetes)<br>40 Diabetes with HbA1c <7.0<br>30 Diabetes with HbA1c 7.0 – 7.9<br>20 Diabetes with HbA1c 8.0 – 8.9<br>10 Diabetes with Hb A1c 9.0 – 9.9<br>0 Diabetes with HbA1c ≥10.0
	**Blood pressure**	**Measurement:** Appropriately measured systolic and diastolic blood pressure	**Metric:** Systolic and diastolic blood pressure (mm Hg)	**Metric:** Systolic and diastolic blood pressure (mm Hg) percentiles for ages 8-12 years. For ages ≥13 years, use adult scoring.<br><br>**Scoring:**

		**Example tools for measurement:**<br>Appropriately sized blood pressure cuff	**Scoring:**<br><u>Points Level</u><br>100 <120/<80 (Optimal)<br>75 120-129/<80 (Elevated)<br>50 130-139 or 80-89 (Stage I HTN)<br>25 140-159 or 90-99<br>0 ≥160 or ≥100<br><br>Subtract 20 points if treated level	<u>Points Level</u><br>100 Optimal (<90th %ile)<br>75 Elevated (≥90th -- <95th %ile or ≥120/80 mm Hg to <95th %ile, whichever is lower)<br>50 Stage I HTN (≥95th -- <95th %ile + 12mmHg, or 130/80 to 139/89 mm Hg, whichever is lower)<br>25 Stage 2 HTN (≥95th %ile + 12mmHg, or ≥140/90 mm Hg, whichever is lower)<br>0 SBP ≥160 or ≥95th %ile + 30mmHg systolic, whichever is lower; and/or DBP ≥100 or ≥95th %ile + 20 mm Hg diastolic<br><br>Subtract 20 points if treated level

*Cannot meet these metrics until solid foods are being consumed
